# Endogenous Molecules Induced by a Pathogen-Associated Molecular Pattern (PAMP) Elicit Innate Immunity in Shrimp

**DOI:** 10.1371/journal.pone.0115232

**Published:** 2014-12-17

**Authors:** Yu-Yuan Chen, Jiann-Chu Chen, Yong-Chin Lin, Suwaree Kitikiew, Hui-Fang Li, Jia-Chin Bai, Kuei-Chi Tseng, Bo-Wei Lin, Po-Chun Liu, Yin-Ze Shi, Yi-Hsuan Kuo, Yu-Hsuan Chang

**Affiliations:** The Center of Excellence for the Oceans, National Taiwan Ocean University, Keelung 202, Taiwan, Republic of China; Uppsala University, Sweden

## Abstract

Invertebrates rely on an innate immune system to combat invading pathogens. The system is initiated in the presence of cell wall components from microbes like lipopolysaccharide (LPS), β-1,3-glucan (βG) and peptidoglycan (PG), altogether known as pathogen-associated molecular patterns (PAMPs), via a recognition of pattern recognition protein (PRP) or receptor (PRR) through complicated reactions. We show herein that shrimp hemocytes incubated with LPS, βG, and PG caused necrosis and released endogenous molecules (EMs), namely EM-L, EM-β, and EM-P, and found that shrimp hemocytes incubated with EM-L, EM-β, and EM-P caused changes in cell viability, degranulation and necrosis of hemocytes, and increased phenoloxidase (PO) activity and respiratory burst (RB) indicating activation of immunity *in vitro*. We found that shrimp receiving EM-L, EM-β, and EM-P had increases in hemocyte count and other immune parameters as well as higher phagocytic activity toward a *Vibrio* pathogen, and found that shrimp receiving EM-L had increases in proliferation cell ratio and mitotic index of hematopoietic tissues (HPTs). We identified proteins of EMs deduced from SDS-PAGE and LC-ESI-MS/MS analyses. EM-L and EM-P contained damage-associated molecular patterns (DAMPs) including HMGBa, HMGBb, histone 2A (H2A), H2B, and H4, and other proteins including proPO, Rab 7 GPTase, and Rab 11 GPTase, which were not observed in controls (EM-C, hemocytes incubated in shrimp salt solution). We concluded that EMs induced by PAMPs contain DAMPs and other immune molecules, and they could elicit innate immunity in shrimp. Further research is needed to identify which individual molecule or combined molecules of EMs cause the results, and determine the mechanism of action in innate immunity.

## Introduction

Like other crustaceans, shrimp rely on innate immunity in which circulating hemocytes play an important role in defending against microbial invasion [1]. Hemocytes are generally classified into three types: hyaline cells (HCs), semi-granular cells (SGCs), and granular cells (GCs) based on their size and degree of granularity [2], [3]. Hemocytes are involved in cellular immune reactions like phagocytosis, nodule formation, and encapsulation and are involved in humoral immune reactions like the prophenoloxidase (proPO) cascade and releases of antimicrobial peptide and lysozymes [4]. The innate immune system is initiated and triggered in the presence of foreign particles, known as pathogen-associated molecular patterns (PAMPs) like lipopolysaccharide (LPS), β-1,3-glucan (βG), and peptidoglycan (PG), that are recognized and bound by pattern recognition proteins (PRPs) or receptors (PRRs) through signaling transduction and degranulation of hemocytes [5]–[7]. Prophenoloxidase (proPO) is cleaved by prophenoloxidase activating enzyme (ppA) or caspase-1-like enzyme into active phenoloxidase (PO), which leads to the formation of melanin [6], [8]. HCs and SGCs are involved in phagocytosis, during which a respiratory burst occurs and leads to the release of the superoxide anion and other reactive oxygen species (ROS) that plays a crucial role in microbicidal activity [9]–[11]. The superoxide anion is the first product during this process, known as a respiratory burst (RB). The proPO activating system and phagocytosis are important immune responses in crustaceans [2], [3]. Several immune molecules and their functions in shrimp immunity have been recently reported [12].

Many scientists reported that crayfish hemocytes and shrimp hemocytes treated with LPS, βG, PG, carrageenan, and fucoidan caused changes in cell size and degranulation of hemocytes *in vitro* [13]–[15]. Furthermore, scientists also reported that shrimp hemocytes incubated in LPS and carrageenan caused changes in cell viability and necrosis *in vitro* [13], [15].

In mammals, cell damage caused by trauma or pathogens releases intracellular molecules or endogenous molecules called damage-associated molecular patterns (DAMPs) or alarmins [16], [17]. They result from cell death due to cell necrosis caused by exogenous PAMPs and play crucial roles in inflammation and activation of innate immunity in mammals [18]–[20]. High mobility group box 1 (HMGB1), a non-histone nuclear protein, is the major DAMPs [18], [20], [21]. However, nothing is known about the presence of endogenous molecules (EMs) associated with cell death of shrimp hemocytes and about the immune response of EMs or DAMPs induced by PAMPs on shrimp.

We assume that shrimp hemocytes receiving LPS, βG, and PG proceed to cell necrosis and release EMs that contain DAMPs and trigger innate immunity. Accordingly, we examined necrosis in hemocytes treated with LPS, βG, and PG and collected the induced EMs, namely EM-L, EM-β, and EM-P, and identified these proteins based on SDS-PAGE and LC-ESI-MS/MS analyses. We examined degranulation, cell viability, and necrosis of hemocytes as well as the immune activation of shrimp hemocytes receiving EM-L, EM-β, and EM-P *in vitro*. We examined the hemocyte count, other immune parameters, and hematopoiesis of hematopoietic tissues (HPTs) of shrimp receiving EMs *in vivo*. Furthermore, we also examined phagocytic activity toward *Vibrio alginolyticus* in shrimp receiving EM-L, EM-β, and EM-P.

## Materials and Methods

### Ethics Statement

We have followed the University's guideline, held the white shrimp *Litopenaeus vannamei*, a species of invertebrate, and conducted the research. The research work and animal holding was approved by the Experimental Animal Center Committee, College of Life Sciences, National Taiwan Ocean University. There is no official recommendation for the experimental use of invertebrates for scientific purpose in Taiwan. The humane killing at the end of the experiment was conducted by completely anaesthetizing the experimental shrimp and then placing them in a freezer to minimize animal suffering.

### Experimental Animal Species

Specific pathogen-free (SPF) white shrimp *L. vannamei*, weighing ∼12 g were obtained from the Aquatic Animal Center, National Taiwan Ocean University. Shrimp were placed in fiberglass tanks (8 m^3^) containing aerated seawater (35‰) and acclimated in the laboratory for two weeks prior to experimentation.

### Experimental Design

Trypsin (bovine pancreatic trypsin), zymosan, LPS (L4005), βG (G5011), and PG (69554) were obtained from Sigma-Aldrich. LPS, βG, and PG were obtained from *Escherichia coli*, *Saccharomyces serevisiae*, and *Bacillus subtilis*, respectively. We conducted eight experiments: (1) observation of cell necrosis in hemocytes incubated with LPS, βG, and PG, (2) identification of EM-L, EM-β, and EM-P induced by LPS, βG, and PG, (3) examination of the effect of EM-L, EM-β, and EM-P on the degranulation of hemocytes, (4) examination of the effect of EM-L, EM-β, and EM-P on cell viability and necrosis of hemocytes, (5) examination of the effects of EM-L, EM-β, and EM-P on the *in vitro* immune activation of shrimp hemocytes, (6) examination of the *in vivo* dynamic effects of EM-L, EM-β, and EM-P on the immune response of shrimp, (7) examination of proliferation cell ratio and mitotic index of hematopoietic tissues (HPTs) of shrimp receiving EM-L, and (8) examination of phagocytic activity in response to *V. alginolyticus* in shrimp receiving EM-L, EM-β, and EM-P.

### Cell Necrosis of Shrimp Hemocytes Incubated with LPS, βG, and PG

Hemolymph (100 µl) was withdrawn from the ventral sinus of each shrimp and diluted with 100 µl of anticoagulant solution (30 mM trisodium citrate, 340 mM sodium chloride, and 10 mM EDTA, at pH 7.5 with the osmolality adjusted to 718 mOsm/kg with 115 mM glucose). LPS, βG and PG solutions were prepared with shrimp salt solution (SSS, anticoagulant solution without EDTA) to make final concentrations of 0.1, 0.5, and 1.0 mg/ml. Details of hemolymph sampling, treatment with LPS, βG and PG, and cell necrosis were conducted following previously described procedures [13]. Briefly, 100 µl of each test solution (LPS, βG, PG) was added to hemolymph sample and incubated for 30 min. A group that 100 µl of SSS was added to hemolymph sample and incubated for 30 min served as controls. The solution was added to 10 µl RNase (1 mg/ml) and incubated for 30 min at 37°C, and 20 µl of propidium iodine (PI, Sigma) solution then added and samples incubated for 15 min at room temperature. Fifty microliters of the mixed solution were spread on a slide glass and centrifuged with cytospin machine (Thermo, Shandon, UK) at 1000 rpm for 3 min. Permanent slides were made and examined under a fluorescent microscope (Model BX 51, Olympus, Tokyo, Japan) for cell death (necrosis) [22]. Hemocytes were also examined under a fluorescent microscope to determine the percentage of necrotic cells.

### Preparation of EM-C, EM-P, EM-β, and EM-P

Briefly, 500 µl of hemolymph was added to a tube containing 500 µl of anticoagulant solution and centrifuged at 800 ×*g* at 4°C for 10 min. The supernatant was discarded and 200 µl of SSS added to make a cell suspension. We then added 100 µl of LPS, βG, and PG solution (1 mg/ml in SSS), and shrimp hemocytes were co-incubated with the solution for 30 min at 4°C to induce cell necrosis. Cell suspensions incubated with 100 µl of SSS served as controls. The incubated solution (300 µl) was centrifuged at 800 ×*g* at 4°C for 10 min. The supernatant containing LPS, βG, or PG was discarded and the precipitated cell pellet rinsed with 100 µl of marine saline (MS, 0.58 M NaCl, 20 mM CaCl_2_, 50 mM Tris-HCl-buffer, 0.6 mM Na_2_HPO_4_, pH 7.4, 718 mOsm/kg) twice to remove residues of LPS, βG, or PG. The precipitated cell pellet was then suspended in 100 µl MS and incubated for 80 min. The incubated solutions were centrifuged at 800 ×*g* at 4°C for 10 min to obtain EMs. The supernatants obtained from SSS, LPS, βG, and PG treatments were named EM-C, EM-L, EM-β, and EM-P, respectively.

### Sodium Dodecyl Sulfate-Polyacrylamide Gel Electrophoresis (SDS-PAGE)

Sodium dodecyl sulfate-polyacrylamide gel electrophoresis (SDS-PAGE) was conducted using polyacrylamide (16%) as the separating gel and 4% polyacrylamide as the stacking gel. Pre-stained protein ladder (Thermo, Waltham, MA, USA) was used as the protein marker. Both Coomassie staining and silver staining were performed.

### Identification of EM-C, EM-L, and EM-P with Liquid Chromatography Electrospray Ionization Tandem Mass Spectrometry (LC-ESI-MS/MS)

After adjusting the pH to 8.5 with 1 M ammonium bicarbonate, 100 µg of total protein were sampled and chemically reduced for 1 h at 60°C by adding dithiothreitol (DTT) to attain 10 mM and carboxyamidomethylated in 55 mM iodoacetamide for 45 min at room temperature in the dark. Trypsin Gold (Promega, Madison, WI, USA) was added to make a final substrate/enzyme ratio of 30:1 (w/w). The trypsin digest was incubated at 37°C for 16 h. After digestion, the peptide mixture was acidified by 10 µl of formic acid (FA) for further mass spectrometry analysis.

After protein digestion, each peptide sample was desalted using a Strata X column (Phenomenex), vacuum-dried, and then resuspended in a 200 µl volume of buffer A (2% acetonitrile (ACN) in 0.1% FA. After centrifugation at 20000 ×*g* for 10 min, the supernatant was recovered to obtain a peptide solution with a final concentration of 0.5 µg/µl. Ten microliters of supernatant were loaded on a LC-20AD nanoHPLC (Shimadzu, Kyoto, Japan) by the autosampler within a 2 cm C18 trap column. Peptides were then eluted into a 10 cm analytical C18 column (inner diameter 75 µm) packed in-house. Samples were loaded at 8 µl/min for 4 min, the 35 min gradient was then run at 300 nl/min starting from 5 to 35% buffer B (95% ACN, 0.1% FA), followed by a 5 min linear gradient to 60%, followed by a 2 min linear gradient to 80%, maintained in 80% buffer B for 4 min, and finally returned to 5% within 1 min.

Data acquisition was performed with a TripleTOF 5600 System fitted with a Nanospray III source (AB SCIEX, Concord, ON, Canada) and a pulled quartz tip as the emitter (New Objectives, Woburn, MA, USA). Data was acquired using an ion spray voltage of 2.5 kV, curtain gas of 30 PSI, nebulizer gas of 15 PSI, and an interface heater temperature of 150°C. Mass spectrometry with a resolving power (RP) of ≥30,000_fwhm_ was used for TOF MS scans. For information-dependent acquisition (IDA), survey scans were acquired in 100 ms and as many as 40 product ion scans were collected if they exceeded a threshold of 150 counts per second (counts/s) and a 2+ to 5+ charge-state. Total cycle time was fixed at 2.8 s. The Q2 transmission window was 80 and 100 Da for 50% light transmission. Four time bins were summed for each scan at a pulse frequency value of 11 kHz through monitoring of the 40 GHz multichannel TDC detector with four-anode/channel detection. Dynamic exclusion was set for 1/2 of the peak width (∼15 s) and the precursor then refreshed from the exclusion list [23]. Data obtained from the LTQ-Orbitrap and 5600 msconverter (Thermo, Waltham, MA, USA) were subjected to protein identification performed by the Mascot Search Engine version 2.3.02 (Matrix Science, London, UK) against a database. A mass tolerance of 0.05 Da was permitted for intact peptide and 0.1 Da for fragmented ions, with allowance for one missed cleavage in trypsin digests. The identified protein was determined using a Blast search program against the non-redundant protein database in NCBI [24].

### Effects of EM-C, EM-L, EM-β, and EM-P on Degranulation

There were four test solutions: EM-C, EM-L, EM-β, and EM-P. Details of hemolymph preparation and hemocytes incubation with each test solution were conducted following previously describe methods [14], [25].

### Effects of EM-C, EM-L, EM-β, and EM-P on Cell Viability and Necrosis of Shrimp Hemocytes

There were four test solutions: EM-C, EM-L, EM-β, and EM-P. Shrimp hemocytes receiving shrimp salt solution (SSS) served as the control group. Cell viability assays were examined at five different incubation times (0, 15, 30, 45, and 60 min) for each test solution. Hemolymph preparation details and procedures for determining cell viability and necrosis were conducted following previously described methods [13], [25], [26], [27].

### Effects of EM-C, EM-L, EM-β, and EM-P on *in vitro* Immune Activation

Phenoloxidase (PO) activity and RB were used as indicators of *in vitro* immune activation. There were eight test solutions consisting of four EM solutions (EM-C, EM-L, EM-β, and EM-P), and four heat-inactivated EM solutions (HEM-C, HEM-L, HEM-β, and HEM-P). The inactivated forms of EM were heated at 80°C (dry bath) for 20 min. Eight shrimp were used in the PO activity assay and another eight were used in the RB assay. In total, there were 10 solutions, including four EMs solutions, four inactivated EMs solutions, one positive control (1 mg/ml trypsin), and one negative control (CAC, background control) in the PO activity assay. For the RB assay, there were 10 solutions similar to those used in the PO activity assay except for one positive control (zymosan), one negative control (MCHBSS). PO activity was measured spectrophotometrically by recording the formation of dopachrome produced from L-dihydroxyphenylalanine (L-DOPA) as previously described [28]. RB was quantified using the reduction of nitroblue tetrazolium (NBT) to formazan to measure the superoxide anion produced [29]. Measurement details are previously described [25].

### Dynamic Effects of EM-C, EM-L, EM-β, and EM-P on Shrimp Immune Response *in vivo*

Immune parameters, including hyaline cells (HCs), granular cells (including semigranular cells, GCs), total hemocyte count (THC), PO activity, RB, superoxide dismutase (SOD) activity, and lysozyme activity were used as immune indicators. There were five test solutions (control, EM-C, EM-L, EM-β, and EM-P) and two exposure times (12 and 24 h). Eight shrimp were injected individually with 20 µl of each EM solution and placed in seawater (35‰). Shrimp injected with 20 µl of marine saline served as controls. Hemolymph sampling and the measurement of immune parameters including HCs, GCs, THC, PO activity, RB, SOD activity, and lysozyme activity were conducted following previously described methods [13]. Immune parameters were normalized for each to the control value (100%), and were presented as relative percent values.

### Effects of EM-L on Proliferation Cell Ratio and the Mitotic Index of HPTs *in vivo*

There was one test solution (EM-L) and one exposure time (24 h). Shrimp were injected individually with 20 µl EM-L and paced in seawater. Shrimp injected with 20 µl of marine saline served as controls. After 24 h, shrimp were then sampled and injected with vinblastine (V1377, Sigma) to inhibit mitosis following previously described methods [25]. Shrimp were dissected and HPTs sampled and fixed with Davidson's fixative solution following the procedures previously described [13]. Details of HPTs sampling, paraffin block preparation, sectioning, staining with hematoxylin and eosin or propidium iodide, and preparation of permanent slides followed procedures previously described [25], [27]. HPTs were observed with optical microscopy (Model BX51, Olympus, Tokyo, Japan). Optical micrographs and fluorescent photographs were both taken. The numbers of proliferating and mitotic cells were counted to establish the percentage of proliferation cells and the mitotic index.

### Effects of EM-C, EM-L, EM-β, and EM-P on Phagocytic Activity in Response to *Vibrio alginolyticus*

Preparation of bacterial suspensions for testing phagocytic activity was conducted following previously described method [10]. There were four EM solutions: EM-C, EM-L, EM-β, and EM-P. Twenty microliters of each EM solution were injected into the third abdominal segment of each tested shrimp. After 24 h, each shrimp was then injected with 20 µl of a bacterial suspension (4.8 × 10^8^ cfu/ml), resulting in 9.6 × 10^6^ cfu per shrimp. Control shrimp received 20 µl of marine saline after 24 h and were then injected with the bacterial suspension. In total, there were five treatments of eight shrimp each. The examination of phagocytic activity (PA) was measured following previously described methods [10], [13].

### Statistical Analysis

Data were subjected to a one-way analysis of variance (ANOVA). Duncan's multiple-comparison test was conducted to examine for significant difference among treatments using SAS computer software (SAS Institute, Cary, NC, USA). A statistically significant difference required that p <0.05.

## Results

### Cell necrosis of Shrimp Hemocytes Caused by LPS, βG, and PG

**Figure 1:**
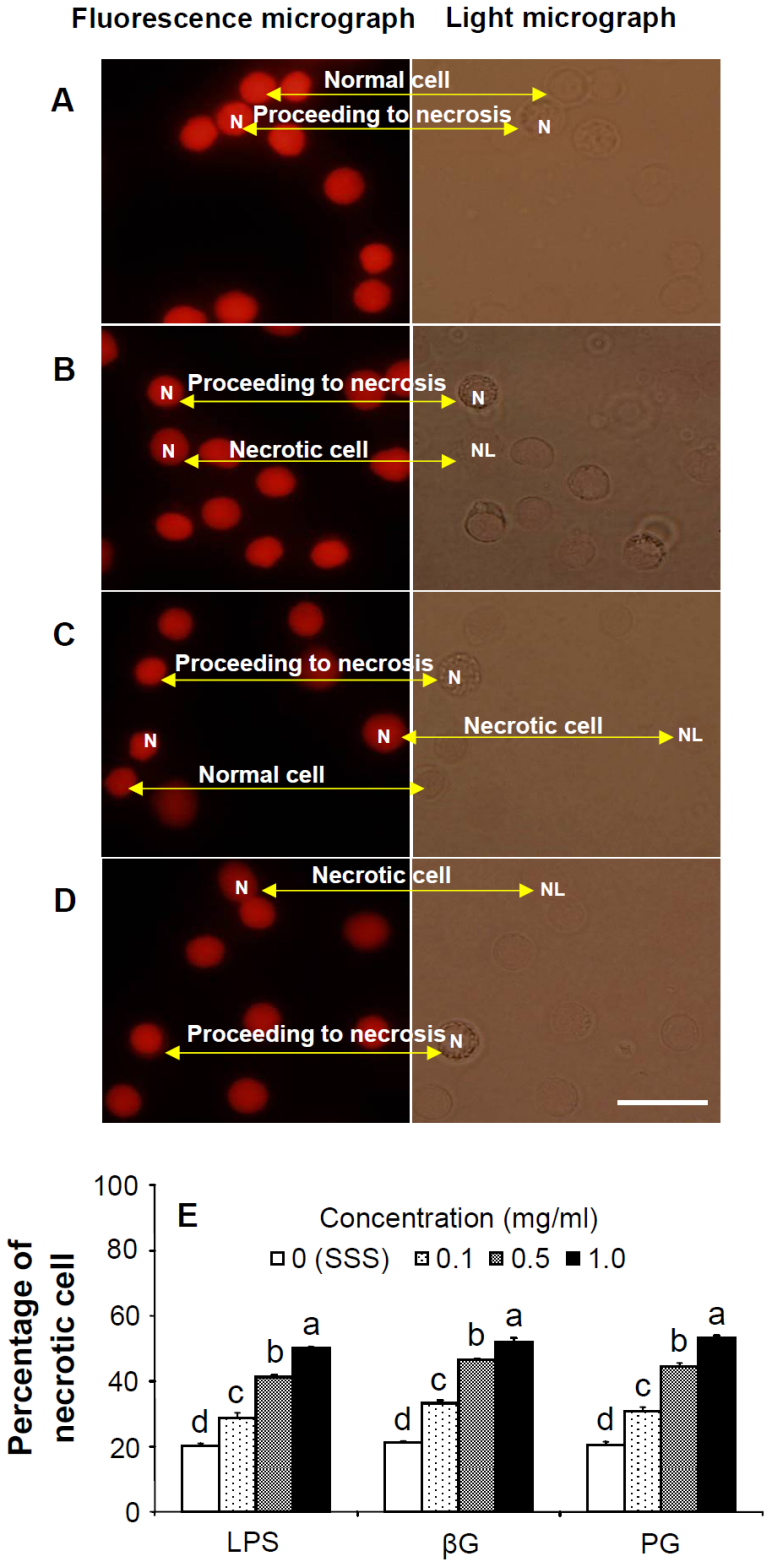
Fluorescence micrographs and light micrographs of shrimp hemocytes incubated in shrimp salt solution (SSS), lipopolysaccharide (LPS), β-1,3-glucan (βG), peptidoglycan (PG) and percentage of necrotic cell. (A) Fluorescence micrographs and light micrographs of shrimp hemocytes incubated in SSS (control) for 30 min. (B) Fluorescence micrographs and light micrographs of shrimp hemocytes incubated in 0.1 mg/ml LPS for 30 min. (C) Fluorescence micrographs and light micrographs of shrimp hemocytes incubated in 0.1 mg/ml βG for 30 min. (D) Fluorescence micrographs and light micrographs of shrimp hemocytes incubated in 0.1 mg/ml PG for 30 min. (E) Percentage of necrotic cells of shrimp hemocytes incubated in SSS (control) and different concentrations (0.1, 0.5, and 1.0 mg/ml) of LPS, βG and PG after 30 min. Each bar represents the mean value from six shrimp with standard error. Data with different letters significantly differ (*p* <0.05) at the same DAMP (LPS, βG, or PG) among different concentrations. Percentages of necrotic cells of hemocytes incubated in 0.1, 0.5, and 1.0 mg/ml LPS, in 0.1, 0.5, and 1.0 mg/ml βG, or in 0.1, 0.5, and 1.0 mg/ml PG were significantly higher than that in controls (SSS). Scale  =  20 µm. N, Nucleus; NL, Nuclei lysed (death cell).

Fluorescence and light micrographs of normal cells, necrosis-proceeding cells, and dead hemocyte cells after 30 min incubation with SSS, LPS, βG, and PG are shown in Fig. 1A-1D. Normal cells have fully intact nuclei, cells proceeding to necrosis have lysed nuclei, and dead cells have no apparent nuclei or DNA. The percentage of cell necrosis in hemocytes incubated with LPS, βG, or PG increased directly with dosage and was significantly higher than in hemocytes incubated with SSS (controls) (Fig. 1E).

### Identification of EM-C, EM-L, EM-β, and EM-P

**Figure 2:**
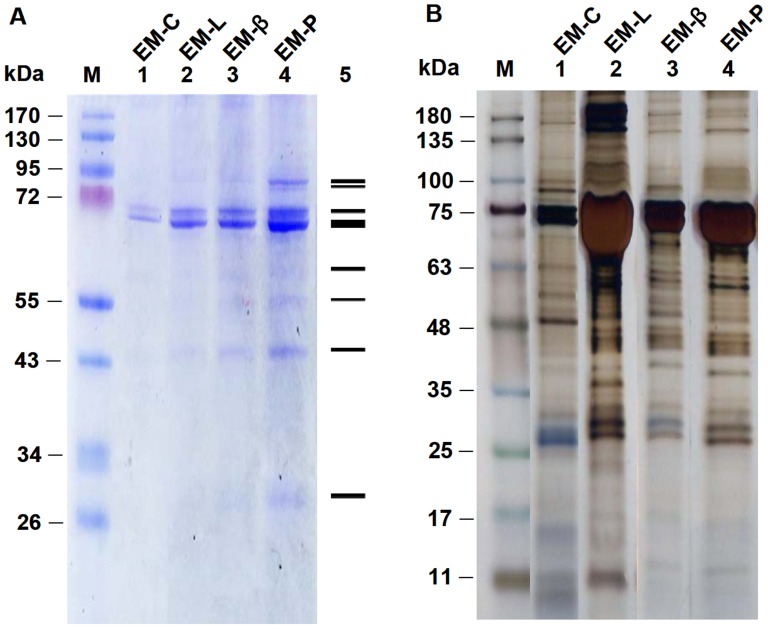
SDS-PAGE analysis of EMs (EM-C, EM-L, EM-β, and EM-P) with Coomassie staining (A) and silver staining (B). Lane M, Molecular mass markers; Lane 1, EM-C; Lane 2, EM-L; Lane 3, EM-β; Lane 4, EM-P. Lane 5, Relative position. EM-C, endogenous molecules of shrimp hemocytes incubated in shrimp salt solution (SSS); EM-L, endogenous molecules of shrimp hemocytes incubated in 1.0 mg/ml lipopolysaccharide (LPS). EM-β, endogenous molecules of shrimp hemocytes incubated in 1.0 mg/ml β-1,3-glucan (βG); EM-P, endogenous molecules of shrimp hemocytes incubated in 1.0 mg/ml peptidoglycan (PG).

EM-C showed two bands (68 and 70 kDa), and EM-L, EM-β, and EM-P showed the same eight bands of proteins with different intensities in Coomassie staining (Fig. 2A). EM-L, EM-β, and EM-P showed about 27–28 bands, and showed two bands between 25–30 kDa in silver-staining (Fig. 2B). An analysis of LC-ESI-MS/MS identified that EM-C, EM-L, and EM-P contained 20, 23, and 20 proteins, respectively (Table 1). EM-L and EM-P contained (1) antimicrobial peptide, (2) proPO system, (3) proteinases/proteinase inhibitors, (4) blood clotting system, (5) signaling transduction, (6) heat shock protein, (7) other immune-related proteins, and (8) DAMPs including HMGBa, HMGBb, histone 2A (H2A), H2B, and H4 with Mascot scores ≥20. However, proPO, Rab 7 GPTase, and Rab 11 GPTase of proPO system and signaling transduction molecules were not observed in EM-C.

**Table 1:** The proteins identified by LC-ESI-MS/MS in the EM-C, EM-L, and EM-P.

No.	Protein identification	Category *	EM-C	EM-L	EM-P	Accession no.	kDa	Score	Coverage (%)
1	Hemolymph clottable protein	4	+	+	+	ABI95361	189.4	8833	33
2	α2-macroglobulin	3	+	+	+	ABI79454	168.6	12316	49
3	Type II transglutaminase (TGs)	4	+	+	+	ABX83902	86.3	6719	48
4	Prophenoloxidase II (proPO II)	2	+	+	+	ABY81277	79.1	6624	49
5	proPO	2	−	+	−	ABL10871	78.4	7723	48
6	proPO I	2	+	+	+	ABX76968	78.4	7417	44
7	Hemocyanin	7	+	+	+	CAA57880	75.0	50090	76
8	TCP-1 Chaperonin	6	+	+	+	AEG80154	58.3	1716	30
9	Caspase 3	3	+	+	+	AGL61582	57.7	340	18
10	Prophenoloxidase activating enzyme (PPAE)	2	+	+	+	AFW98991	51.3	382	20
11	Serine proteinase inhibitor	3	+	+	+	AGZ91893	46.4	1832	40
12	Mas trypsin-like serine proteinase	3	+	+	+	AFW98990	38.7	1948	35
13	KPI2	3	+	+	+	AFK25798	25.8	4776	59
14	HMGBa	8	+	+	+	ADQ43366	25.6	259	18
15	Kazal-type proteinase inhibitor (KPI)	3	+	+	+	AAT09421	25.5	7643	70
16	HMGBb	8	+	+	+	ADQ43367	24.0	1174	32
17	Rab 7 GTPase	5	−	+	−	AFD54570	23.4	32	6
18	Rab GTPase	5	+	+	+	AFK08607	23.2	413	28
19	Anti-lipopolysaccharide factor (ALF)	1	+	+	+	ABB22832	13.9	110	13
20	Histone 2A (H2A)	7, 8	+	+	+	Q6PV61	13.3	430	32
21	H2B	7, 8	+	+	+	P83863	12.8	538	21
22	H4	7, 8	+	+	+	P83865	11.4	518	42
23	Rab 11 GTPase	5	−	+	+	AER34937	10.9	27	9

*1.Antimicrobial peptides, 2.proPO system, 3. proteinase/proteinase inhibitors, 4.blood clotting system, 5.signaling transduction, 6.heat shock proteins, 7.other immune molecules, 8.damage-associated molecular pattern (DAMP) [12], [18], [19].

EM-C, shrimp hemocytes incubated in shrimp salt solution (SSS); EM-L, Shrimp hemocytes incubated in polysaccharide (LPS). EM-P, Shrimp hemocytes incubated in peptidoglycan (PG).

### Effect of EM-C, EM-L, EM-β, and EM-P on Degranulation

**Figure 3:**
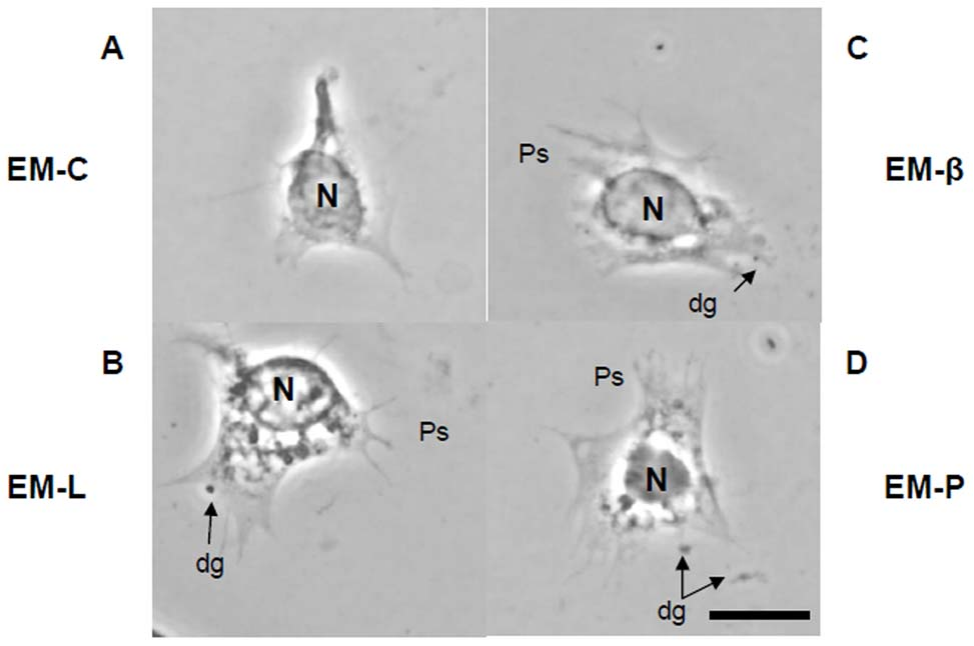
Monolayer of shrimp hemocytes incubated in EM-C, EM-L, EM-β, EM-P. (A) Monolayer of shrimp hemocytes in EM-C. (B) Monolayer of shrimp hemocytes incubated in EM-L. (C), Monolayer of shrimp hemocytes incubated in EM-β. (D) Monolayer of shrimp hemocytes incubated in EM-P. dg, degranulation; Ps, pseudopodia; Scale  =  10 µm. EM-C, endogenous molecules of shrimp hemocytes incubated in shrimp salt solution (SSS); EM-L, endogenous molecules of shrimp hemocytes incubated in 1.0 mg/ml lipopolysaccharide (LPS). EM-β, endogenous molecules of shrimp hemocytes incubated in 1.0 mg/ml β-1,3-glucan (βG); EM-P, endogenous molecules of shrimp hemocytes incubated in 1.0 mg/ml peptidoglycan (PG).

Monolayers of granular cells after 30 min of incubation with EM-C, EM-L, EM-β, and EM-P are shown in Fig. 3. Granular cells incubated with EM-L, EM-β, and EM-P showed degranulation.

### Cell Viability and Necrosis of Hemocytes Incubated with EM-C, EM-L, EM-β, and EM-P

**Figure 4:**
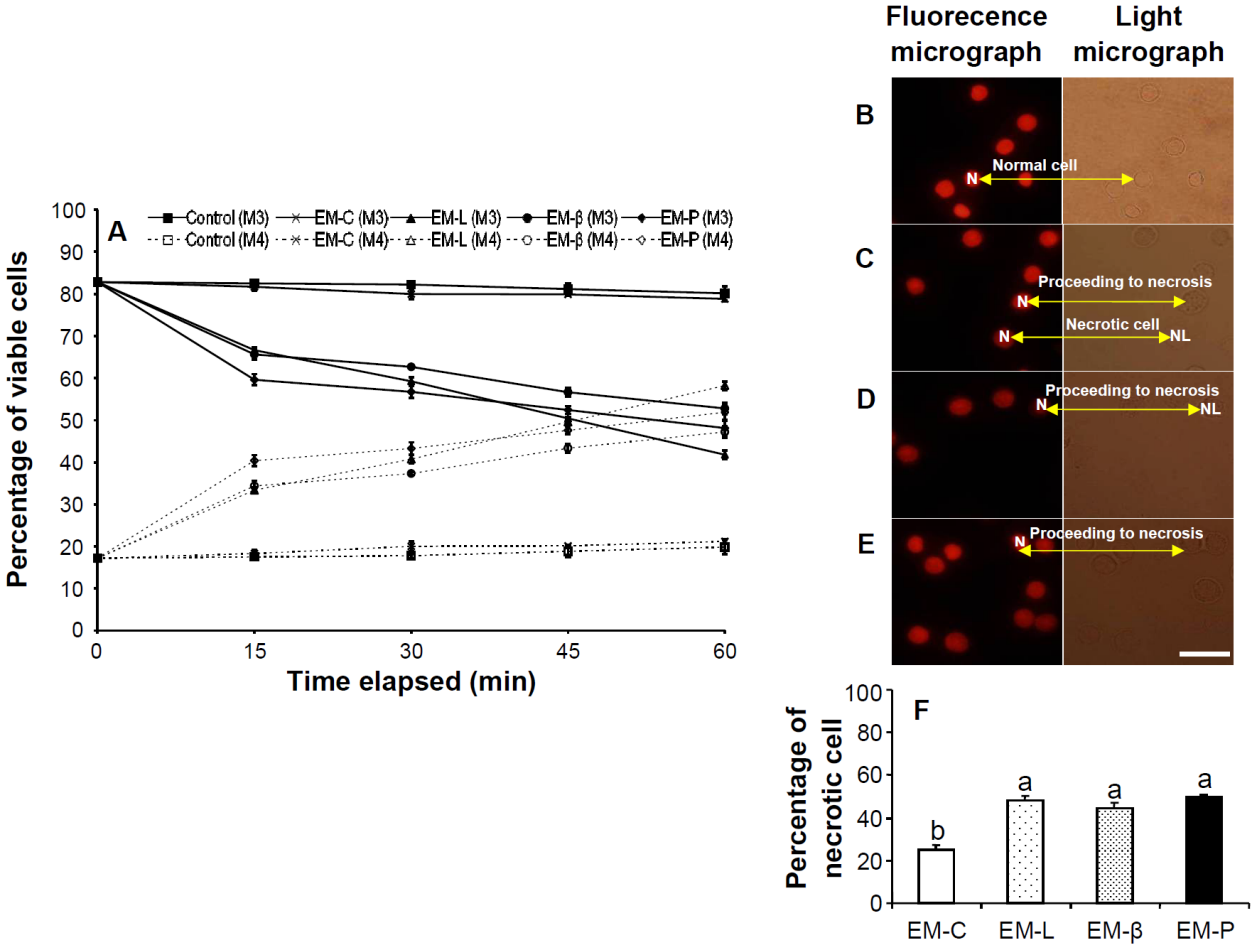
Changes in cell viability and necrosis of shrimp hemocytes incubated in EM-C, EM-L, EM-β, and EM-P, and percentage of necrotic cell of different EMs. (A) Viable and nonviable cells are designated in marker 3 (M3) and marker 4 (M4), respectively. Changes in viability over time of shrimp hemocytes showing the kinetics and proportion of viable hemocyte cells incubated in EM-C, EM-L, EM-β, and EM-P. Cell viability of shrimp hemocytes receiving shrimp salt solution (SSS) served as the control group. (B–E) Fluorescence micrographs and light micrographs showing necrosis of shrimp hemocytes incubated in EM-C (B), EM-L (C), EM-β (D), and EM-P (E) for 30 min. (F) Percentage of necrotic cells of shrimp hemocytes incubated in EM-C, EM-L, EM-β, and EM-P for 30 min. Each bar represents the mean value from six shrimp with standard error. Data with different letters significantly differ (*p* <0.05) among treatments. The percentages of necrotic cells of shrimp hemocytes incubated in EM-L, EM-β, and EM-L were significantly higher than in EM-C. See Fig. 2 for the abbreviation of EM-C, EM-L, EM-β, and EM-P. N, Nucleus; NL, Nuclei lysed (dead cells); Scale  =  20 µm.

The percentage of live cells remained constant at 80%–83% and 79%–83% in hemocytes incubated with SSS (control) and EM-C, respectively. However, in shrimp hemocytes incubated with EM-L, EM-β, and EM-P, the percentage of live cells decreased over time to 42%–53% after 60 min with no significant differences among the three treatments (Fig. 4A). Fluorescence and light micrographs of normal cells, necrosis-proceeding cells, and dead cells after 30 min incubation in EM-C, EM-L, EM-β, and EM-P are shown in Fig. 4B, 4C, 4D, and 4E, respectively. Normal cells have fully intact nuclei, cells proceeding to necrosis have lysed nuclei, and dead cells have no apparent nuclei or DNA. The percentages of cell necrosis in hemocytes incubated with EM-L, EM-β, and EM-P were significantly higher than in hemocytes incubated with EM-C (Fig. 4F).

### Effects of EM-C, EM-L, EM-β, and EM-P on Immune Activation *in vitro*

**Figure 5:**
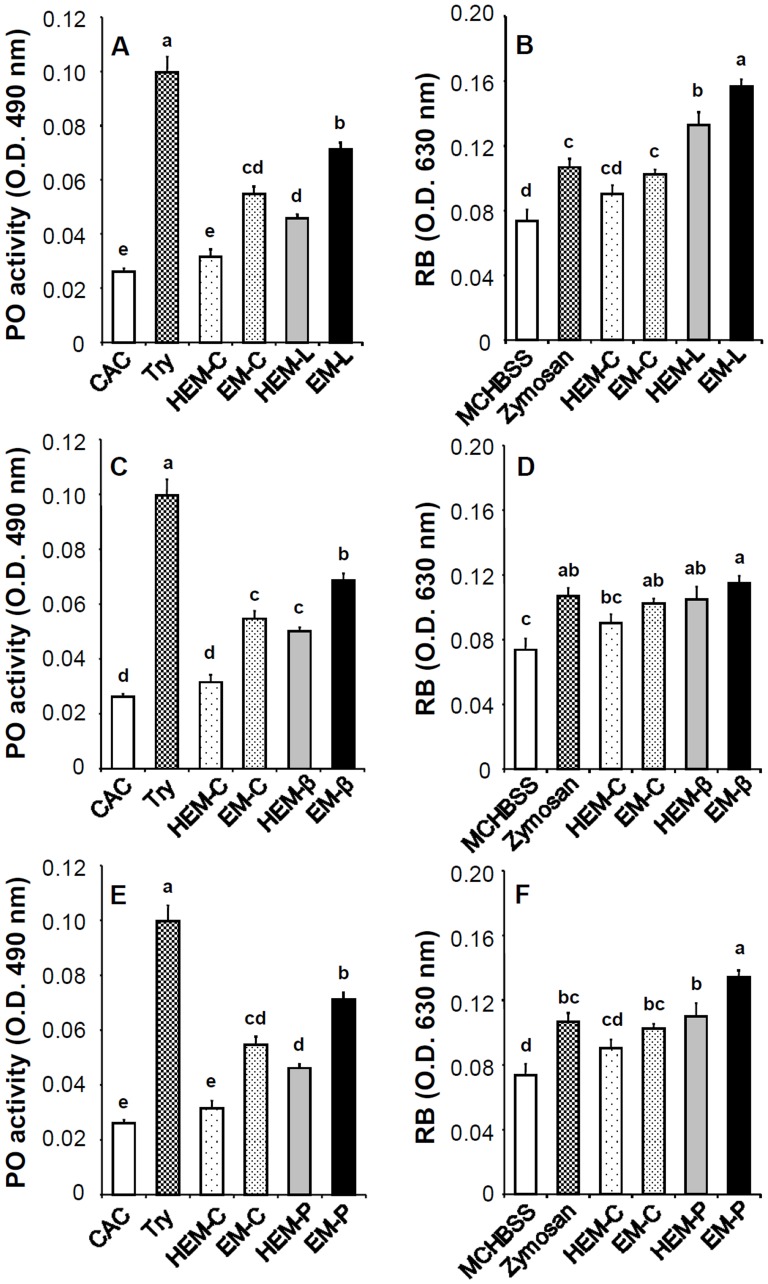
Phenoloxidase (PO) activity and respiratory burst (RB, release of superoxide anion) of shrimp hemocytes incubated with EM-C, EM-L, EM-β and EM-P. (A) PO activity of shrimp hemocytes incubated in heat-inactivated EM-C (HEM-C), EM-C, heat-inactivated EM-L (HEM-L), and EM-L. (B) RB of shrimp hemocytes incubated in HEM-C, EM-C, HEM-L, and EM-L. (C) PO activity of shrimp hemocytes incubated in HEM-C, EM-C, heat-inactivated EM-β (HEM-β), and EM-β. (D) RB of shrimp hemocytes incubated in HEM-C, EM-C, HEM-β, and EM-β. (E) PO activity of shrimp hemocytes incubated in HEM-C, EM-C, heat-inactivated EM-P (HEM-P), and EM-P. (F) RB of shrimp hemocytes incubated in HEM-C, EM-C, HEM-P, and EM-P. CAC served as negative control for the PO activity assay and MCHBSS served as the negative control for the RB assay. Trypsin (Try) served as the positive control for PO activity assay and zymosan served as the positive control for RB assay. See Fig. 2 for the abbreviation of EM-C, EM-L, EM-β, and EM-P. Each bar represents the mean value from eight shrimp with standard error. Data in the same parameter with different letters significantly differ (*p* <0.05) among treatments.

PO activity in shrimp hemocytes incubated with EM-L, EM-β, and EM-P was significantly higher than in shrimp hemocytes incubated with HEMs (HEM-L, HEM-β, and HEM-P), EM-C, HEM-C, and CAC (Fig. 5A, 5C, 5E). The RB of hemocytes incubated with EM-L was significantly higher than in shrimp hemocytes incubated with HEM-L, EM-C, HEM-C, and MCHBSS. Similarly, the RB of hemocytes incubated in EM-P and EM-β were significantly higher than that in shrimp hemocytes incubated in HEM-C and MCHBSS (Fig. 5B, 5D, 5F).

### Dynamic Effects of EM-C, EM-L, EM-β, and EM-P on Shrimp Immune Response *in vivo*

**Figure 6:**
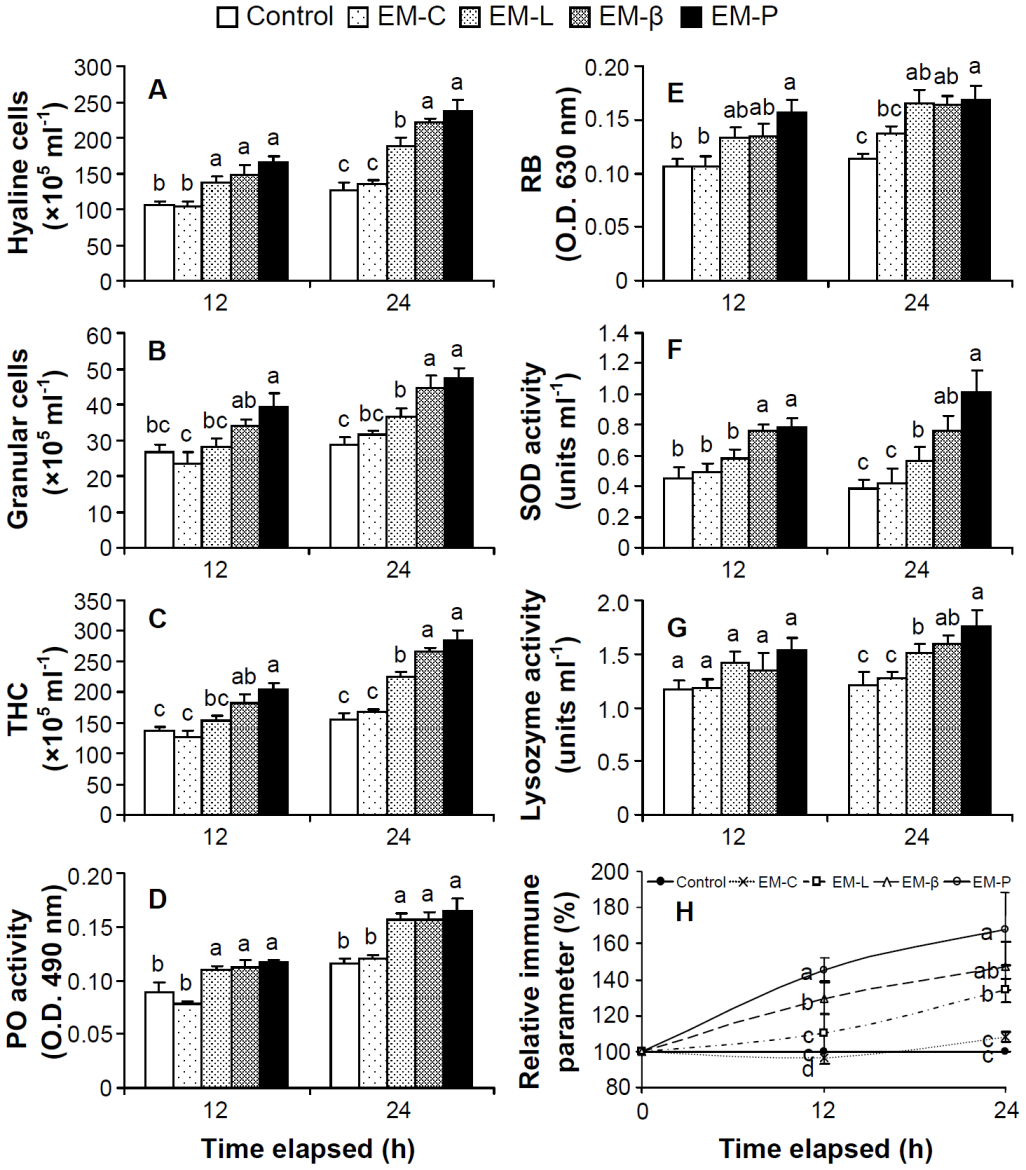
The immune parameters of white shrimp *Litopenaeus vannamei* that received EM-C, EM-L, EM-β, and EM-P after 12 and 24 h. The immune parameters of shrimp without any treatment served as the control group. (A) hyaline cells (HCs); (B) granular cells (including semigranular cells, GCs); (C) total hemocyte count (THC); (D) phenoloxidase activity; (E) respiratory burst (RB, release of superoxide anion); (F) superoxide dismutase (SOD) activity; (G) lysozyme activity; (H) relative immune parameter (%) of control shrimp, and shrimp receiving EM-C, EM-L, EM-β, and EM-P after 12 and 24 h. See Fig. 2 for the abbreviation of EM-C, EM-L, EM-β, and EM-P. Each bar represents the mean value from eight shrimp with standard error. Data in the same parameter and same time with different letters significantly differ (*p* <0.05) among treatments.

The immune parameters of shrimp receiving EM-L, EM-β, and EM-P increased with time. Shrimp receiving EM-L, EM-β, and EM-P all had significantly higher HCs, GCs, THC, PO activity, RB, SOD activity, and lysozyme activity, compared to controls after 24 h (Fig. 6A–6G). Shrimp receiving EM-P, EM-β, and EM-L exhibited significantly increased relative immune parameters by 168%, 147%, and 133%, compared to controls (Fig. 6H).

### Effects of EM-L on Proliferation Cell Ratio and the Mitotic Index of HPTs *in vivo*

**Figure 7:**
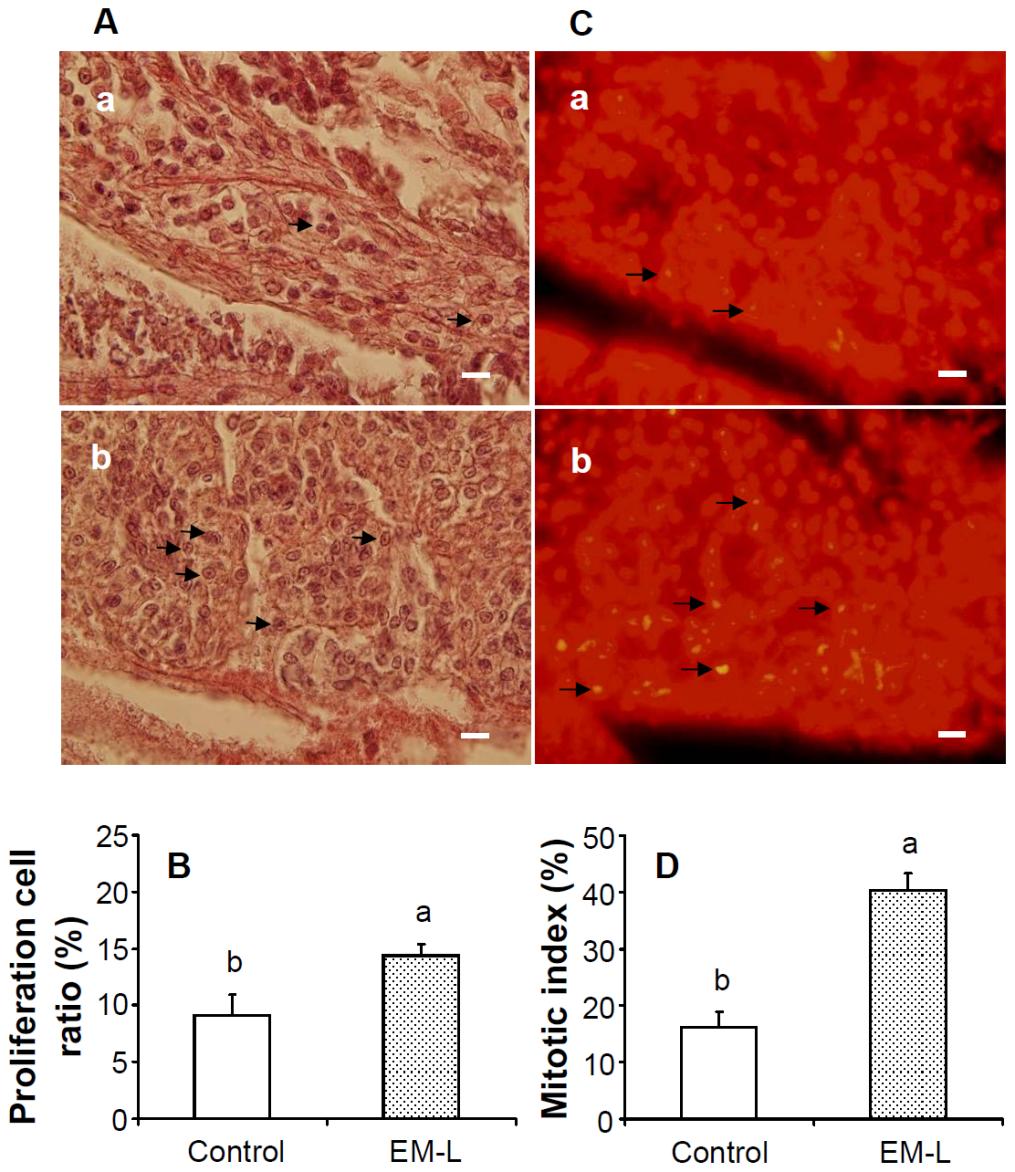
Cell proliferation cell ratio (%) and mitotic index (%) in hematopoietic tissues (HPTs). (A) Optical micrograph of H&E-stained HPTs of control shrimp (a) and shrimp receiving EM-L (b) after 24 h. A higher number of mitotic cells (arrows) were observed in shrimp receiving EM-L. (B) Cell proliferation cell ratio (%) in HPTs of control shrimp and shrimp receiving EM-L. (C) Fluorescence micrograph of propodium iodide-stained HPTs of control shrimp (a), and shrimp receiving EM-L (b) after 24 h. A higher number of mitotic cells (arrows) were observed in shrimp receiving EM-L. (D) Mitotic index (%) in HPTs of control shrimp and shrimp receiving EM-L. Scale  =  20 µl. Each bar represents the mean value from ten images with standard error. Data in the same parameter with different letters significantly differ (*p* <0.05) between treatments. Shrimp received marine saline served as control shrimp. See Fig. 2 for the abbreviation of EM-L.

Optical photographs of H&E-stained HPTs of shrimp receiving EM-C and EM-L after 24 h are shown in Fig. 7A. The proliferation cell ratio of shrimp receiving EM-L was significantly higher than that of EM-C (Fig. 7B). Fluorescence photographs of PI-stained HPTs of shrimp receiving EM-C and EM-L are shown in Fig. 7C. The mitotic index of shrimp receiving EM-L was significantly different from that of EM-C (Fig. 7D).

### Effects of EM-C, EM-L, EM-β, and EM-P on Phagocytic Activity toward *V. alginolyticus*

**Figure 8:**
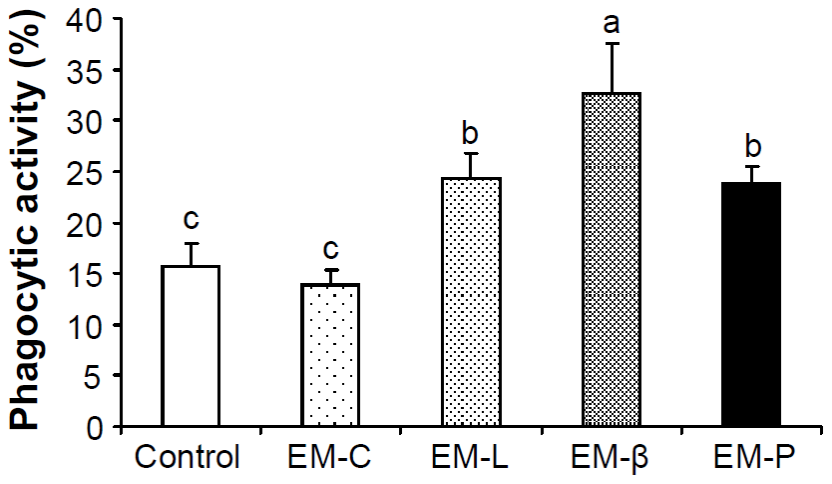
Phagocytic activity of shrimp that received EM-C, EM-L, EM-β, and EM-P after 24 h and then received *Vibrio alginolyticus* challenge. Phagocytic activity of shrimp receiving marine saline after 24 h and then receiving *V. alginolyticus* served as the control group. Phagocytic activity of shrimp receiving EM-L, EM-β, and EM-P was significantly higher than in control and shrimp receiving EM-C. Each bar represents the mean value from eight shrimp with standard error. See Fig. 2 for the abbreviation of EM-C, EM-L, EM-β, and EM-P. Data with different letters significantly differ (*p* <0.05) among treatments.

Phagocytic activity in shrimp receiving EM-L, EM-β, and EM-P was significantly higher than in control shrimp and shrimp receiving EM-C (Fig. 8).

## Discussion

In mammals, some endogenous molecules known as DAMPs induced by PAMPs are released during cell death and stimulate inflammation [17], [30]. High-mobility group box B1 or high-mobility group protein B1 (HMGB1), HSPs, and histones are important DAMPs [19]. HMGB family comprises three members, HMGB1, HMGB2, and HMGB3, in mammals and bony and cartilaginous fish, and these three proteins are more than 80% identical at the amino acid level [31]. HMGB1 appears at variable levels in most cells and is released by cells undergoing non-apoptotic (unscheduled) cell death [16]. HMGB1, a 25 kDa protein, is considered to be a prototypic DAMP [18], [19]. In the present study, HMGBa and HMGBb with molecular weights of 25.6 kDa and 24.0 kDa, respectively, were observed in EM-C, EM-L, and EM-P (Table 1). A multiple alignment of HMGBa and HMGBb in white shrimp was similar to that of HMGB1 and HMGB2 in other species with the presences of two DNA binding domains (A box and B box) as well as tail (S1 Table, S1 Figure). The expression of HMGB1 (1a and 1b) and HMGB2 (2a and 2b) in grass carp are significantly up-regulated by viral PAMPs, poly-inosinic-polycytidylic potassium salt (poly(I:C)), bacterial PAMPs, LPS, and PG challenges in CIK cells [32], [33]. Therefore, it is suggested that the release of HMGBa and HMGBb occurs naturally during cell necrosis and occurs in shrimp hemocytes in response to LPS, βG and PG.

HSPs function as molecular chaperones, assisting in the correct folding or refolding of nascent and misfolded proteins. HSPs are secreted actively, released passively by necrotic cells, and are involved in active nonclassical secretion and play a role in inflammation/immunity [16]. Histone 2B (H2B) phosphorylation of DNA causes tight binding in HMGB1 and chromosomes, subsequently preventing HMGB1 release into the extracellular space between cells undergoing apoptosis [18]. In addition, caspase is an important effecter protein and mediates the apoptotic process [34], and caspase 3 (AGL61582) is known to be involved in the release of HMGB1 [19]. In the present study, TCP-1 chaperonin of heat shock protein, H2A, H2B and H4 of DAMPs, caspase 3 of proteinase, and other molecules were present in EM-L and EM-P. The functions of these molecules involved in the DAMPs are needed for further study.

It is known that SGCs and GCs are induced to degranulate by LPS, βG, and fucoidan, and subsequently release proPO activation system proteins like proPO and peroxinectin, and antimicrobial peptides and transglutaminase, indicating activation of innate immunity [2], [12], [13], [14], [35]. Scientists also indicate that that proPO, peroxinectin, PPAE, proteinase inhibitors, antimicrobial peptides, clotting proteins and integrin are released from live cells [2], [6], [12]. In addition, lysis occurred in crayfish *Astacus astacus* hemocytes treated with βG [36]. Necrosis occurred in shrimp hemocytes incubated with carrageenan [13]. Similarly, necrosis occurred in shrimp hemocytes incubated with LPS, βG, and PG in the present study. Furthermore, shrimp hemocytes incubated with EM-L, EM-β, and EM-P (induced endogenously by LPS, βG, and LPS) all caused changes in cell viability and degranulation and immune activation in terms of increases in PO activity and RB indicating activation of innate immunity. It is suggested that during the process of necrosis of hemocyte in response to foreign particles, dying cells release molecules and leave a danger signal to alert other hemocytes to elicit immune activation. In this study, about 20-23 hemocyte proteins were observed. However, we are not sure which ones are from live cells and which ones are originating from disintegrated dead cells, in addition to the above mentioned molecuels. Further research is needed to clarify this problem.

Scientists have indicated that the formation of the DAMP-PAMP complex with HMGB may trigger innate immunity [17], [30]. Scientists have also indicated the possible function of DAMPs (HMGB1, HSPs) and PAMPs with their pattern-recognition receptors (PRRs) that are involved in the danger signal, and indicated that HMGB1 can bind to LPS and induce immune responses [37 38]. In the present study, about 20–23 proteins were observed in EMs. HMGBa, HMGBb and histones (H2A, H2B, and H4) were observed in EM-L and EM-P. However, proPO of proPO system, and Rab 7 GPTase and Rab 11 GPTase of signaling transduction molecules were not observed in EM-C (Table 1). This fact indicated that the EM-L and EM-P induced endogenously by LPS and PG contained some molecules that were absent in EM-C. Despite some molecules being observed in EM-C, EM-L, EM-β, and EM-P, the quantities of each molecule may differ. For instance, two clear protein bands (68 and 70 kDa) appeared in EM-L, EM-β and EM-P with much higher protein quantity in Coomassie staining. Furthermore, two bands (25–30 kDa) appeared in EM-L, EM-B, and EM-P with higher protein quantity in silver staining. It is suggested that EMs containing DAMPs and other immune related proteins are released naturally into hemolymph in background levels without affecting alerting. However, more EMs containing DAMPs and immune related proteins are released by LPS, βG or PG-induced necrosis, and they trigger immune activation.

In the present study, we found that the EM-L, EM-β, and EM-P induced activation of innate immunity *in vitro* and increased immune parameters of shrimp and its resistance against *V. alginolyticus in vivo*. Therefore, we are sure that EM-L, EM-β, and EM-P induced by LPS, βG, and PG endogenously caused the activation of immunity and immune response. However, we are not sure which single molecule or combined molecules caused the results. We are not sure whether the formation of the DAMP-PAMP complex occurs and whether DAMPs bind to their PRRs and activate innate immunity similar to those in HMGB1 [17], [37], [38]. Further research is needed to identify different bands obtained, and produce recombinant of each molecule like HMGB and conduct *in vitro* and *in vivo* experiments.

**Figure 9:**
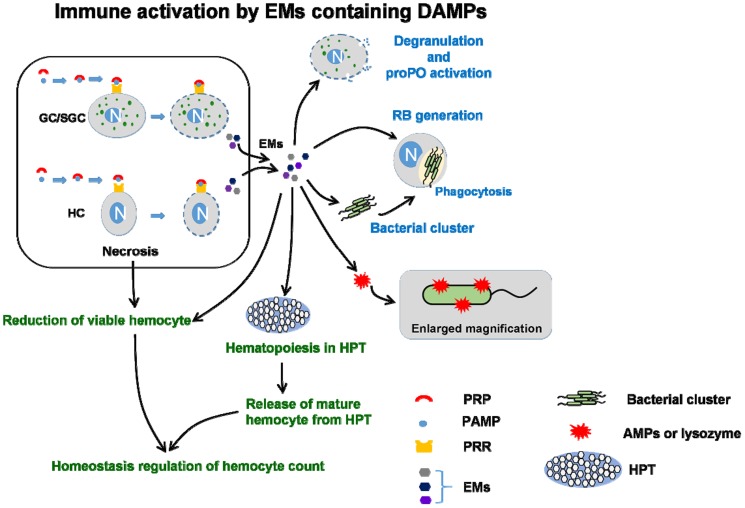
Diagram showing the immune activation of endogenous molecules (EMs) induced by lipopolysaccharide (LPS), β-1,3-glucan (βG), and peptidoglycan (PG). EMs contains DAMPs including HMGBa, HMGB, and other DAMPs (H2a, H2b, H4), signaling transduction molecules (Rab 7 GTPase, Rab 11 GPTase) and other immune molecules.

We like to make further discussion. In the present study, we found that EM-L, EM-β, and EM-P contained mixed molecules. The immune parameters of shrimp receiving EM-L, EM-β, and EM-P increased directly with time. HCs, GCs, THC, PO activity, RB, SOD activity, and lysozyme activity significantly increased in shrimp receiving EM-L, EM-β, and EM-P after 24 h. HCs, GCs, THC, PO activity, RB, SOD activity, and lysozyme activity in shrimp receiving EM-L increased by 78%, 38%, 63%, 76%, 53%, 26%, and 29%, respectively, after 24 h along with higher proliferation cell ratio and mitotic index of HPTs. The phagocytic activity of shrimp receiving EM-L, EM-β, and EM-P and then challenged with *V. alginolyticus* was significantly higher than in EM-C and controls. Therefore, EM-L, EM-β, and EM-P can trigger immunity, increase immune response of shrimp and its increase resistance against *Vibrio* pathogen. Here, we propose a model where EMs containing DAMPs and other immune molecules activate the innate immune system (Fig. 9).

In conclusion, PAMPs including LPS, βG, and PG caused the necrosis of hemocytes, leading to release of EMs containing DAMPs like HMGB (a prototype of DAMP), H2A, H2b, H4, signaling transduction molecules (Rab 7 GTPase and Rab 11 GPTase), and other molecules. HMGBa and HMGBb from shrimp were highly similar to HMGB1 and HMGB2 from mammals, amphibians, teleosts, and insects. Shrimp hemocytes incubated in EM-L, EM-β, and EM-P activate innate immunity as evidenced by increases in PO activity and RB *in vitro*. Shrimp receiving EM-L, EM-β, and EM-P had increased hemocyte counts and other parameters as well as higher phagocytic activity in reaction to a *Vibrio* pathogen *in vivo*. This study documents that the EMs of shrimp hemocytes induced by LPS, βG, and PG endogenously contain DAMPs and other immune-related proteins, and that EMs elicit innate immunity. Further research is needed to examine single molecule like HMGB or the combined mixtures of EMs, and determine their mechanisms of action in shrimp's innate immunity.

## Supporting Information

S1 Figure A multiple alignment of HMGBa and HMGBb in white shrimp was similar to that of HMGB1 and HMGB2 in other species with the presences of two DNA binding domains as well tail. Multiple alignment of LV-HMGBa (ADQ43366), LV-HMGBb (ADQ43367), HS-HMGB1 (CAG33144), MM-HMGB1 (AAI10668), XL-HMGB1 (NP_001080836), CI-HMGB1a (34), CI-HMGB1b (34), CI-HMGB2a (33), CI-HMGB2b (33), DR-HMGB1a (AAH45917), DR-HMGB1b (NP_001092721), DR-HMGB2a (NP_001032501), DR-HMGB2b (NP_001004674), DV-putative HMG-like (AAO92280), MO-HMG-DSP1-like (XP_003746359), and PH-HMG-B2 (XP_002422686).
(DOC)

S1 Table Species, abbreviation of proteins, GenBank accession numbers of HMGB sequences, and identity and similarity analyses.
(DOC)

## References

[1] Hoffman JA, Kafatoa FC, Janeway CA Jr, Ekekowitz RAB (1999) Phylogenetic properties in innate immunity. Science 284:1313–1318. 10334979 10.1126/science.284.5418.1313

[2] Jiravanichpaisal P, Lee BL, Söderhäll K (2006) Cell-mediated immunity in arthropods: Hematopoiesis, coagulation, melanization and opsonization. Immunobiology 211:213–236. 16697916 10.1016/j.imbio.2005.10.015

[3] Rowley AF, Powell A (2010) Invertebrate immune system specific, quasi-specific, or nonspecific? J Imunol 179:7209–7214. 10.4049/jimmunol.179.11.7209 18025161

[4] Loker ES, Adema CM, Zhang SM, Kepler TB (2004) Invertebrate immune system-not homogenous, not simple, not well understood. Immunol Rev 198:10–24. 15199951 10.1111/j.0105-2896.2004.0117.x PMC5426807

[5] Janeway CA Jr, Medzhitov CA (2002) Innate immune recognition. Annu Rev Immunol 20:197–216. 11861602 10.1146/annurev.immunol.20.083001.084359

[6] Cerenius L, Lee BL, Söderhäll K (2008) The proPO-system: pros and cons for its role in invertebrate immunity. Trends Immunol 29:263–71. 18457993 10.1016/j.it.2008.02.009

[7] Amparyup P, Charoensapsri W, Tassanakajon A (2013) Prophenoloxidase system and its role in shrimp immune responses against major pathogen. Fish Shellfish Immunol 34:990–1001. 22960099 10.1016/j.fsi.2012.08.019

[8] Jearaphunt M, Noonin C, Jiravanichpaisal P, Nakamura S, Tassanakajon A, et al (2014) Caspase-1-like regulation of the proPO-system and role of ppA Caspase-1-like cleaved peptides from proPO in innate immunity. PLoS Pathog 10:e1004059. 24722332 10.1371/journal.ppat.1004059 PMC3983073

[9] Giulianini PG, Bierti M, Loreszon S, Battistella S, Antonio E (2007) Ultrastructural and functional characterization of circulating hemocytes from the freshwater crayfish *Astacus leptodactylus*. Cell types and their role after *in vivo* artificial non-self challenge. Micron 38:49–57. 16839768 10.1016/j.micron.2006.03.019

[10] Lin YC, Chen JC, Morni WZ, Putra DF, Huang CL, et al (2013) Vaccination enhances early immune responses in white shrimp *Litopenaeus vannamei* after secondary exposure to *Vibrio alginolyticus* . PLoS ONE 8:e69722. 23894531 10.1371/journal.pone.0069722 PMC3718771

[11] Beutler B (2004) Innate immunity: an overview. Mol Immunol 40:845–859. 14698223 10.1016/j.molimm.2003.10.005

[12] Tassanakajon A, Somboonwiwat K, Supungul P, Tang S (2013) Discovery of immune molecules and their crucial functions in shrimp immunity. Fish Shellfish Immunol 34:954–967. 23059654 10.1016/j.fsi.2012.09.021

[13] Chen YY, Chen JC, Lin YC, Putra DF, Kitikiew S, et al (2014) Shrimp that received carrageenan via immersion and diet exhibit immunocompetence in phagocytosis despite a post-plateau in immune parameters. Fish Shellfish Immunol 36:352–366. 24361621 10.1016/j.fsi.2013.12.004

[14] Smith VJ, Söderhäll K (1983) β-1,3-glucan activation of crustacean hemocyte *in vitro* and *in vivo* . Biol Bull 164:299–314.

[15] Xian JA, Wang AL, Tian JX, Huang JW, Ye CX, et al (2009) Morphologic, physiological and immunological changes of haemocytes from *Litopenaeus vannamei* treated by lipopolysaccharide. Aquaculture 298:139–145.

[16] Bianchi ME (2007) DAMPs, PAMPs and alarmins: all we need to know about danger. J. Leukocyte Biol 81:1–5. 10.1189/jlb.0306164 17032697

[17] Pisetsky DS (2011) Cell death in the pathogenesis of immune-mediated diseases: The role of HMGB1 and DAMP-PAMP complexes. Swiss Med Wkly 141:w13256. 21877298 10.4414/smw.2011.13256 PMC3724463

[18] Vera-Jimenz NI, Nielsen ME (2013) Carp head kidney leukocytes display different patterns of oxygen radical production after stimulation with PAMPs and DAMPs. Mol Immunol 55:231–236. 23517739 10.1016/j.molimm.2013.01.016

[19] Li G, Tang D, Lotze MT (2013) Ménage à Trosi in stress: DAMPS, redox and autophagy. Seminar in Cancer Biol 23:380–390. 10.1016/j.semcancer.2013.08.002 PMC4085481 23994764

[20] Lotze MT, Zeh HJ, Rubartelli A, Sparvero LJ, Amoscato AA, et al (2007) The grateful dead: damage-associated molecular pattern molecules and reduction/oxidation regulate immunity. Immunol Rev 220:60–81. 17979840 10.1111/j.1600-065X.2007.00579.x

[21] Lotze MT, Tracey KJ (2005) High-mobility group box 1 protein (HMGB1): nuclear weapon in the immune arsenal. Nature Rev Immunol 5:331–342. 15803152 10.1038/nri1594

[22] Rieger AM, Nelson KL, Konowalchuk JD, Barreda DR (2011) Modified annexin v/propidium iodide apoptosis assay for accurate assessment of cell death. J Vis Exp 50:e2597. 10.3791/2597 PMC3169266 21540825

[23] Andrew CL, Simons BL, Young JB, Hawkridge AM, Muddiman DC (2011) Performance characteristics of a new hybrid quadrupole time-of-flight tandem mass spectrometer (TripleTOF 5600). Anal Chem 83:5442–5446. 21619048 10.1021/ac200812d PMC3138073

[24] Gazzola D, Vincenzi S, Gastaldon L, Tolin S, Pasini G, et al (2014) The proteins of the grape (*Vitis vinifera L.*) seed endosperm: Fractionation and identification of the major components. Food Chemistry 155:132–139. 24594165 10.1016/j.foodchem.2014.01.032

[25] Kitikiew S, Chen JC, Putra DF, Lin YC, Yeh ST, et al (2013) Fucoidan effectively provokes the innate immunity of white shrimp *Litopenaeus vannamei* and its resistance against experimental *Vibrio alginolyticus* infection. Fish Shellfish Immunol 34:280–290. 23201320 10.1016/j.fsi.2012.11.016

[26] Cárdenas W, Dankert JR, Jenkins JA (2004) Flow cytometric analysis of crayfish haemocytes activated by lipopolysaccharides. Fish Shellfish Immunol 17:223–233. 15276602 10.1016/j.fsi.2003.03.001

[27] van de Braak CBT, Botterblom MHA, Liu W, Taverne N, van der Knaap WPW, et al (2002) The role of the haematopoietic tissue in haemocyte production and maturation in the black tiger shrimp (*Penaeus monodon*). Fish Shellfish Immunol 12:253–272. 11931020 10.1006/fsim.2001.0369

[28] Hernández-López J, Gollas-Galván TS, Vargas-Albores F (1996) Activation of the phenoloxidase system of the brown shrimp (*Penaeus californiensis* Holmes). Comp Biochem Physiol Part C, Pharmacol Toxicol Endocrinol 113:61–66.

[29] Bell KL, Smith VJ (1993) *In vitro* superoxide production by hyaline cells of the shore crab. *Caranus maenas* (L.) and its resistance against *Vibrio alginolyticus* . Dev Comp Immunol 17:211–219. 8392007 10.1016/0145-305x(93)90040-w

[30] Zheng Y, Gardner SE, Clarke MCH (2011) Cell death, damage-associated molecular patterns, and sterile inflammation in cardiovascular disease. Arterioscler Throm Vasc Biol 31:2781–2786. 10.1161/ATVBAHA.111.224907 22096097

[31] Moleri S, Cappellano G, Gaudenzi G, Cermenati S, Cotelli F, et al (2011) The HMGB protein gene family in zebrafish: Evolution and embryonic expression patterns. Gene Exp Patterns 11:3–11. 10.1016/j.gep.2010.08.006 20804857

[32] Rao Y, Su J, Yang C, Peng L, Feng X, et al (2013) Characterization of two grass carp *Ctenopharyngodon idella* HMGB2 genes and potential roles in innate immunity. Dev Comp Immunol 43:164–177. 10.1016/j.dci.2013.06.002 23756189

[33] Yang C, Peng L, Su J (2013) Two HMGB1 genes from grass carp *Ctenopharyngodon idella* mediate immune response to variable/bacterial PAMPs and GCRV challenge. Dev Comp Immunol 39:133–146. 23228458 10.1016/j.dci.2012.11.008

[34] Wang L, Zhi B, Wu W, Zhang X (2008) Requirement for shrimp caspase in apoptosis against virus infection. Dev Comp Immunol 32:706–715. 18068223 10.1016/j.dci.2007.10.010

[35] Johansson MW, Lind MI, Holmbald T, Thörnqvist PO, Söderhäll K (1995) Peroxinectin, a novel cell adhesion protein from crayfish blood. Biochem Biophys Res Comm 216:1079–1086. 7488183 10.1006/bbrc.1995.2731

[36] Smith VJ, Söderhäll K (1983) Induction of degranulation and lysis of haemocytes in the freshwater crayfish, *Astacus astacus* by components of the prophenoloxidase activating system *in vitro* . Cell Tissue Res 233:295–303. 6413069 10.1007/BF00238297

[37] Dubaniewicz A (2013) Microbial and human heat shock proteins as ‘danger signals’ in sarcoidosis. Human Immunol 74:1550–1558. 23993988 10.1016/j.humimm.2013.08.275

[38] Pisetsky DS (2012) The origin and properties of extracellular DNA: from PAMP to DAMP. Clin Immunol 144:32–40. 22659033 10.1016/j.clim.2012.04.006 PMC3724456

