# Supplementary material for: Endogenous Molecules Induced by a Pathogen-Associated Molecular Pattern (PAMP) Elicit Innate Immunity in Shrimp
Source: PLoS One. 2014 Dec 17;9(12):e115232. doi: 10.1371/journal.pone.0115232 (PMC4269435; doi:10.1371/journal.pone.0115232)
Supplement: S1 Figure — A multiple alignment of HMGBa and HMGBb in white shrimp was similar to that of HMGB1 and HMGB2 in other species with the presences of two DNA binding domains as well tail. Multiple alignment of LV-HMGBa (ADQ43366), LV-HMGBb (ADQ43367), HS-HMGB1 (CAG33144), MM-HMGB1 (AAI10668), XL-HMGB1 (NP_001080836), CI-HMGB1a (34), CI-HMGB1b (34), CI-HMGB2a (33), CI-HMGB2b (33), DR-HMGB1a (AAH45917), DR-HMGB1b (NP_001092721), DR-HMGB2a (NP_001032501), DR-HMGB2b (NP_001004674), DV-putative HMG-like (AAO92280), MO-HMG-DSP1-like (XP_003746359), and PH-HMG-B2 (XP_002422686). (DOC) [file pone.0115232.s001.doc]

**Figure S1**

A-box (71 aa)

1 100

LV_HMGBa (1) --------MPRGRPRGVAAEKPRGRMTAYAFFVQTCRTEHKKLHPDENVQFAEFSRQCSERWKTMSDKEKKKFHDMAEDDKKRYDEEMKDFVPSPGAGRR

LV_HMGBb (1) --------MPRAKLVDS---KPRGRMSAYAFFVQTCREEHKKKHPDENVVFSEFSRKCAERWKTMTDKEKDRFYDMADKDKARYDTEMKGYR-GPRTPRV

HS_HMGB1 (1) -------------MGKGDPKKPRGKMSSYAFFVQTCREEHKKKHPDASVNFSEFSKKCSERWKTMSAKEKGKFEDMAKADKARYEREMKTYIPPK--GET

MM_HMGB1 (1) -------------MGKGDPKKPRGKMSSYAFFVQTCREEHKKKHPDASVNFSEFSKKCSERWKTMSAKEKGKFEDMAKADKARYEREMKTYIPPK--GET

XL_HMGB1 (1) -------------MGKGDPKKPRGKMSSYAYFVQTCREEHKKKHPDASVNFAEFSKKCSERWKTMSAKEKSKFEDMAKADKVRYEREMKTYIPPK--GET

CI_HMGB1a (1) -------------MGK-DPTKPRGKMSSYAYFVQTCREEHKKKHPEATVNFSEFSKKCSERWKTMSAKEKGRFEDMAKLDKARYEREMKNYIPPK--GEK

CI_HMGB1b (1) -------------MGK-DPRKPKGKMSSYAYFVQTCREEHKKKHPEATVNFSEFSKKCSERWKTMSAKEKGKFEDMAKQDKVRFEREMKNYIPPK--GEK

CI_HMGB2a (1) --------------MGKDTNKPRGKTSSYAFFVQTCREEHKKKNPGTAVNFAEFSKKCSERWKTMSSKEKGKFEEMAKTDKVRYDREMKNYVPPK-GAKG

CI_HMGB2b (1) -------------MVKGDVNKPKGKTSAYAYFVQTCRDEHKRKSPDVPVNFSEFSKKCSERWKSLNASEKSKFEDLAKADKVRYDKEMKNYVPPKGVGKA

DR_HMGB1a (1) -------------MGK-DPTKPRGKMSSYAYFVQTCREEHKKKHPEATVNFSEFSKKCSERWKTMSAKEKGKFEDMAKLDKARYEREMKNYIPPK--GEK

DR_HMGB1b (1) -------------MGK-DPRKPRGKMSSYAYFVQTCREEHKKKHPEASVNFSEFSKKCSERWKTMSAKEKGKFEDMAKQDKVRYEREMKNYIPPK--GEK

DR_HMGB2a (1) --------------MGKDPNKPRGKTSSYAFFVQTCREEHKKKNPGTSVNFSEFSKKCSERWRTMSSKEKGKFEEMAKTDKVRYDREMKNYVPPK-GAKG

DR_HMGB2b (1) -------------MVKGDVNKPKGKTSAYAFFVQTCRDEHKRKSPDVPVNFSEFSKKCSERWKSLNASDKVKFEDMAKADKVRYDREMKTYVPPKGVGKT

DV_putative HMG-like (1) ---------------MGKGDKPRGRMSAYAFFVQTCREEHKKKHPNENVVFAEFSKKCAERWKTMSESEKKRFHQMADKDKKRFDTEMADYK--PPKGDK

MO_HMG-DSP1-like (1) MLIETNLSDPKLELKMAKGDKPRGRMSAYAYFVQTCREEHKKKHPNENVVFAEFSKKCAERWKTMNEPEKQRFHLMAAKDKKRYENEMSTYV--PKEGPR

PH_HMG-B2 (1) --------MPRGKN----DNKPRGRMTAYAFFVQTCREEHKKKHPDENVVFAEFSKKCAERWKTMLDKEKRRFHEMAEKDKLRYDSEMQNYVPSKAE-KR

Consensus (1) M K DP KPRGKMSSYAFFVQTCREEHKKKHPD SVNFSEFSKKCSERWKTMSAKEKGKFEDMAK DKVRYDREMKNYIPPK GEK

B-box (69 aa)

101 200

LV_HMGBa (93) GRRARGRRPKDPNKPKRALSAFFYYANDERPKVRAANPDFSVGEVAKELGRQWNELGEDEKVKYEKLAEEDRARYDREMTAYKFGGASPQKKMK-AS---

LV_HMGBb (89) SRKRR--NRKDPNAPKRALSAFFWFCNDERAKVRAANPDMGVGDVAKQLGAAWSNTPPEAKAKYEALAASDKERYEKEMKAFK--EGNFGAKKH-KT---

HS_HMGB1 (86) KKKFK-----DPNAPKRPPSAFFLFCSEYRPKIKGEHPGLSIGDVAKKLGEMWNNTAADDKQPYEKKAAKLKEKYEKDIAAYRAKG-KPDAAKKGVVKAE

MM_HMGB1 (86) KKKFK-----DPNAPKRPPSAFFLFCSEYRPKIKGEHPGLSIGDVAKKLGEMWNNTAADDKQPYEKKAAKLKEKYEKDIAAYRAKG-KPDAAKKGVVKAE

XL_HMGB1 (86) KKKFK-----DPNAPKRPPSAFFLFCSEFRPKIKGEHPGSTIGDIAKKLGEMWNNTATDDKLPFERKAAKLKEKYEKDVAAYRAKG-KPEPAKKAPAKPE

CI_HMGB1a (85) KKRFK-----DPNAPKRPPSAFFIFCAEFRPKVKEETPGLSIGDVAKKLGEMWNKTSSEEKQPYEKKAAKLKEKYEKDIAAYRSKG-KVGGATAKAP---

CI_HMGB1b (85) KRRFK-----DPNAPKRPPSAFFIFCGDYRPKIRGENPGLSIGDIAKKLGEMWNSSSAEVKQPYEKKAAKLKEKYDKDIALYRTKG-IAGLSKKDG----

CI_HMGB2a (86) GKKKK-----DPNAPKRPPSAFFVFCSDHRPKVKNDNPGISIGDIAQKLGEMWSKLSPKEKAPYEQKAMKLKEKYEKEVAAYRAKGAKVDGGKKGGPGRP

CI_HMGB2b (88) GRKKK-----DPNAPKRPPSAFFVFCSEYRPTVKSENPGLSIGETAKKLGEMWSKQGIKDRAPFEQKAMKLREKYEKDVAAYRAG----GGASKRGPGRP

DR_HMGB1a (85) KKRFK-----DPNAPKRPPSAFFIFCSEFRPKVKEETPGLSIGDVAKRLGEMWNKISSEEKQPYEKKAAKLKEKYEKDIAAYRSKG-KVGGGAAKAP---

DR_HMGB1b (85) KKRFK-----DPNAPKRPPSAFFIFCGDYRPKIKGENPGLSIGDIAKKLGEMWNSSSAEVKQPYEKKAAKLKEKYDKDIALYRTKG-IAGFSKKEG----

DR_HMGB2a (86) GKKKK-----DPNAPKRPPSAFFVFCSDHRPKVKGDNPGISIGDIAKKLGEMWSKLSPKEKSPYEQKAMKLKEKYEKDVAAYRAKGVKPDGAKKGGPGRP

DR_HMGB2b (88) GRKKK-----DPNAPKRPPSAFFVFCSEYRPTVKSEHPNLTIGEIAKKLGELWSKQSSKDRAPFEQKAGKLREKYEKEVAAYRAG----GGASKRGPGRP

DV_putative HMG-like (84) SKKRK--RAKDPNAPKRPLSAFFWFCNDERPNVRQESPDASVGEVAKELGRRWNEVGDDVKSKYEGLAAKDKARYEKELKAYK--GKKPKAASPPKE---

MO_HMG-DSP1-like (99) GKAAR--KKKDPNAPKRALSAFFHFCQDERPKVKATLGESTVAEVAKELGRKWQDCTDEQKGKYEQLAAKDKQRYEREMTAYKKGGVVPAAPEP------

PH_HMG-B2 (88) GKKRK--HIKDPNAPKRSLSAFFWFCNDERPKVKAINPEYGVGDVAKELGRRWAEADPDTKSKYEAMAEKDKARYDRK--------K-------------

Consensus (101) KK K DPNAPKRPPSAFFIFCSDYRPKVKGENPGLSIGDVAKKLGEMWN S DDK PYEKKAAKLKEKYEKDIAAYRAKG K GGAKK G

Tail (26 aa)

201 238

LV_HMGBa (189) ----NGHPVDADPEDDEDEVGGEEDDEDDVSDEGSEDE

LV_HMGBb (181) ----MNAPNEDDDEEEESESEEEEEEEDDE--------

HS_HMGB1 (180) KSKKKKEEEEDEEDEEDEEEEEDEEDEDEEEDDDDD--

MM_HMGB1 (180) KSKKKKEEEDDEEDEEDEEEEEEEEDEDEEEDDDDE--

XL_HMGB1 (180) KAKKK--EEDDEDDDEEDEDEEDEEEEEEEDDDE----

CI_HMGB1a (176) ---SKPDKVNDEDDDDNDEEEEDDDDDDDEDDE-----

CI_HMGB1b (175) --------GEDDDENEDEE-EEEEEEEDEEDDE-----

CI_HMGB2a (181) AG--KKVEADDDDDDDEEEEDEEEEDEEDEDDDDD---

CI_HMGB2b (179) AGSTKKVQPDDDDDDDDDEEDEDEEEDEDEDDDDE---

DR_HMGB1a (176) ---SKPDKANDEDEDDDEEEDEDDDDEEEDDDE-----

DR_HMGB1b (175) --------GEDDEEDEDDD-EEEDDEEEEDDE------

DR_HMGB2a (181) AG--KKAEADDDDDEDEDEEDEEEEDEEDEDDDDE---

DR_HMGB2b (179) TGSVKKSQAEADDDDDEDEDEEDEEDDEEEEDEDDE--

DV_putative HMG-like (177) ----KAKKKEEDDDEDDDDEEDEVEDAEDDDDDDED--

MO_HMG-DSP1-like (191) ---------EYDDDDDEDDDE-----------------

PH_HMG-B2 (165) -------PKSFADDDDEEELEEDDEEDEDM--------

Consensus (201) K EDDDDDDEDEEEEEEEDEEDEDDDD
