# Supplementary material for: Endogenous Molecules Induced by a Pathogen-Associated Molecular Pattern (PAMP) Elicit Innate Immunity in Shrimp
Source: PLoS One. 2014 Dec 17;9(12):e115232. doi: 10.1371/journal.pone.0115232 (PMC4269435; doi:10.1371/journal.pone.0115232)
Supplement: S1 Table — Species, abbreviation of proteins, GenBank accession numbers of HMGB sequences, and identity and similarity analyses. (DOC) [file pone.0115232.s002.doc]

**Table S1.**

| Species | Abbreviation | Accession no. / | Size | MW | Identity **(%)** | Identity (%) |
| --- | --- | --- | --- | --- | --- | --- |
|  |  | Reference | (aa) | (kDa) | Similarity (%) | Similarity (%) |
| **Vertebrate** |  |  |  |  |  |  |
| **Mammalia** |  |  |  |  |  |  |
| *Homo sapiens* | HMGB1 | CAG33144 | 215 | 24.9 | 45.5, 62.6 | 49.8, 62.9 |
| *Mus musculus* | HMGB1 | AAI10668 | 215 | 24.9 | 44.9, 62.6 | 49.8, 62.9 |
| *Otolemur garnettii* | HMGB1 | XP_003797684 | 289 | 32.3 | 33.6, 46.5 | 37.3, 47.1 |
| **Amphibia** |  |  |  |  |  |  |
| *Xenopus laevis* | HMGB1 | NP_001080836 | 211 | 24.5 | 43.5, 62.3 | 49.3, 62.7 |
| **Teleost** |  |  |  |  |  |  |
| *Ctenopharyngodon idella* | HMGB1a | [33] | 205 | 23.7 | 44.6, 59.9 | 50.0, 63.2 |
| *C.idella* | HMGB1b | [33] | 198 | 23.1 | 44.1, 59.0 | 50.0, 65.0 |
| *C.idella* | HMGB2a | [32] | 213 | 24.1 | 45.8, 58.7 | 49.3, 62.6 |
| *C.idella* | HMGB2a | [32] | 213 | 24.1 | 41.3, 60.5 | 45.2, 61.8 |
| *Danio rerio* | HMGB1a | AAH45917 | 205 | 23.7 | 46.4, 59.9 | 51.4, 64.6 |
| *D. rerio* | HMGB1b | NP_001092721 | 197 | 23.1 | 43.2, 58.6 | 49.0, 64.6 |
| *D. rerio* | HMGB2a | NP_001032501 | 213 | 24.1 | 44.4, 59.6 | 48.4, 62.6 |
| *D. rerio* | HMGB2b | NP_001004674 | 214 | 24.3 | 42.9, 59.8 | 45.4, 62.4 |
| **Invertebrate** |  |  |  |  |  |  |
| **Nematoda** |  |  |  |  |  |  |
| *Caenorhabditis elegans* | HMG-1.2, isoform b | NP_001022599 | 234 | 27.2 | 33.7, 45.6 | 37.2, 47.7 |
| **Chelicerata** |  |  |  |  |  |  |
| *Amblyomma variegatum* | putative HMG-like | DAA34592 | 145 | 17.0 | 44.6, 51.4 | 46.1, 53.4 |
| *Dermacentor variabilis* | putative HMG-like | AAO92280 | 208 | 24.2 | 56.4, 67.1 | 56.8, 69.5 |
| *Metaseiulus occidentalis* | HMG-DSP1-like isoform 3 | XP_003746359 | 202 | 23.5 | 49.6, 62.6 | 53.0, 64.1 |
| **Crustacea** |  |  |  |  |  |  |
| *Litopenaeus vannamei* | HMGBa | ADQ43366 | 222 | 25.6 | Present study | 55.4, 66.5 |
| *L. vannamei* | HMGBb | ADQ43367 | 206 | 24.0 | 55.4, 66.5 | Present study |
| **Insecta** |  |  |  |  |  |  |
| *Pediculus humanus corporis* | putative HMGB2 | XP_002422686 | 187 | 22.3 | 52.3, 66.2 | 58.7, 71.4 |
| **Pismire** |  |  |  |  |  |  |
| *Harpegnathos saltator* | HMG-DSP1 | EFN75836 | 445 | 49.4 | 24.0, 30.7 | 26.9, 31.8 |
| *Acromyrmex echinatior* | HMG-DSP1 | EGI65660 | 305 | 34.7 | 33.8, 43.4 | 39.7, 46.5 |
| Camponotus floridanus | HMG-DSP1 | EFN74839 | 305 | 33.8 | 34.6, 43.0 | 39.2, 46.0 |
| **Mosquito** |  |  |  |  |  |  |
| *Anopheles darlingi* | HMG-DSP1 | ETN66707 | 494 | 57.2 | 22.6, 28.9 | 23.9, 29.1 |
| **Fly** |  |  |  |  |  |  |
| *Ceratitis capitata* | HMG-DSP1-like | XP_004529459 | 392 | 44.5 | 29.0, 35.9 | 30.6, 36.7 |
| *Drosophila melanogaster* | DNA-binding protein | AAA50238 | 393 | 45.5 | 29.1, 35.7 | 29.8, 36.9 |
| **Bee** |  |  |  |  |  |  |
| *Apis dorsata* | HMG-DSP1-like | XP_006618174 | 420 | 47.4 | 26.2, 31.9 | 28.7, 33.8 |
| *Apis mellifera* | HMG-DSP1 | XP_001121384 | 378 | 42.7 | 21.6, 26.7 | 24.7, 28.9 |
| *Apis florae* | HMG-DSP1-like | XP_003696576 | 420 | 47.5 | 26.2, 31.9 | 28.7, 33.8 |
| *Bombus impatiens* | HMG-DSP1-like | XP_003486555 | 456 | 51.4 | 24.2, 29.5 | 26.5, 31.2 |
| *Nasonia vitripennis* | HMG-DSP1 | XP_001605958 | 433 | 49.1 | 26.1, 33.0 | 27.4, 33.6 |
